# PKA and PKC Balance in Synapse Elimination during Neuromuscular Junction Development

**DOI:** 10.3390/cells10061384

**Published:** 2021-06-04

**Authors:** Neus Garcia, Maria A. Lanuza, Marta Tomàs, Víctor Cilleros-Mañé, Laia Just-Borràs, Maria Duran, Aleksandra Polishchuk, Josep Tomàs

**Affiliations:** Unitat d’Histologia i Neurobiologia (UHNEUROB), Facultat de Medicina i Ciències de la Salut, Universitat Rovira i Virgili, Sant Llorenç 21, 43201 Reus, Spain; mariaangel.lanuza@urv.cat (M.A.L.); marta.tomas@urv.cat (M.T.); victorcilleros@gmail.com (V.C.-M.); l.just.borras@gmail.com (L.J.-B.); maria.duran@urv.cat (M.D.); aleksandra.polishchuk@estudiants.urv.cat (A.P.)

**Keywords:** motor end-plate, postnatal synapse elimination, axonal competition, acetylcholine release, nicotinic acetylcholine receptor clusters, protein kinases, PKC, PKA

## Abstract

During the development of the nervous system, synaptogenesis occurs in excess though only the appropriate connections consolidate. At the neuromuscular junction, competition between several motor nerve terminals results in the maturation of a single axon and the elimination of the others. The activity-dependent release of transmitter, cotransmitters, and neurotrophic factors allows the direct mutual influence between motor axon terminals through receptors such as presynaptic muscarinic ACh autoreceptors and the tropomyosin-related kinase B neurotrophin receptor. In previous studies, we investigated the synergistic and antagonistic relations between these receptors and their downstream coupling to PKA and PKC pathways and observed a metabotropic receptor-driven balance between PKA (stabilizes multinnervation) and PKC (promotes developmental axonal loss). However, how much does each kinase contribute in the developmental synapse elimination process? A detailed statistical analysis of the differences between the PKA and PKC effects in the synapse elimination could help to explore this point. The present short communication provides this analysis and results show that a similar level of PKA inhibition and PKC potentiation would be required during development to promote synapse loss.

## 1. Introduction and Methods

We published recently in *Cells* a research article entitled: Opposed Actions of PKA Isozymes (RI and RII) and PKC Isoforms (cPKCβI and nPKCε) in Neuromuscular Developmental Synapse Elimination (by Garcia, N.; Balañà, C.; Lanuza, M.A.; Tomàs, M.; Cilleros-Mañé, V.; Just-Borràs, L.; Tomàs, J. Cells 2019, 8, 1304) (PMID: 31652775) in which we pursued previous investigations on the molecular mechanisms involved in the developmental synapse elimination topic. The present short communication highlights details on the statistical analysis of the differences between the PKA and PKC effects on axonal competition and the synapse loss process.

During the development of the nervous system, synapses formed in excess though only the appropriate connections consolidate. At the neuromuscular junction (NMJ), competition between several motor nerve terminals results in the maturation of only one axon and the elimination of the others [1,2]. The activity-dependent release of ACh, adenosine, and neurotrophic factors among other molecules allows the direct mutual influence between motor axon terminals and neurons with the involvement of the postsynaptic muscle and teloglial cells [3,4,5]. Thus, the competitive signaling between motor axons is supported, among other receptors, by presynaptic muscarinic ACh autoreceptors (mAChR: M_1_, M_2_, and M_4_ types), adenosine receptors (AR: A_1_ and A_2A_), and the tropomyosin-related kinase B neurotrophin receptor (TrkB). In previous studies, we investigated the synergistic and antagonistic relations between these receptors that affect synapse elimination [6,7]. Receptors A_1_, M_1_, and TrkB operate mainly through the protein kinase C (PKC) pathway whereas A_2A_, M_2_, and M_4_ are coupled to the protein kinase A (PKA) pathway [8]. In the forementioned paper [9], we described that PKA-I and II activity seems to stabilize multiinervation by delaying both axonal elimination and postsynaptic nicotinic ACh receptors (nAChR) pretzel-like cluster differentiation in P5-P9 neonatal mice. Contrarily, PKC activity promotes both developmental axon loss (through cPKCβI and nPKCε isoform action) and postsynaptic nAChR cluster maturation (a possible role for PKCθ). Thus, a metabotropic receptor-driven balance between PKA and PKC activities in the competing axon terminals and, probably, in the postsynaptic site, could be relevant in developmental synapse elimination phenomenom. The phosphorylation of pre- and postsynaptic PKA and PKC targets involved in transmitter release and nerve terminal and/or nAChRs stability could realize the final molecular mechanism of synapse loss.

Therefore, to further know the PKA and PKC action on this mechanism, we ask several questions that can be answered by comparing PKA and PKC effects on the nerve terminal loss and nAChRs cluster maturation: What can be the relative contribution of each kinase in developmental synapse elimination? What is more determinant to final synapse elimination: PKA inhibition or PKC activation? A detailed statistical analysis of the differences between the PKA and PKC effects in the synapse elimination process will help to explore these crucial points and encourage more specific experiments. Here we provide this analysis by using the data of the previous paper in *Cells*. Experiments were performed on the Levator auris longus (LAL) muscle from P9 transgenic B6.Cg-Tg (Thy1-YFP)16 Jrs/J mice. In summary, subcutaneous injections of solutions (Table 1) were administered on the LAL external surface as previously described [10,11].

**Figure 1:**
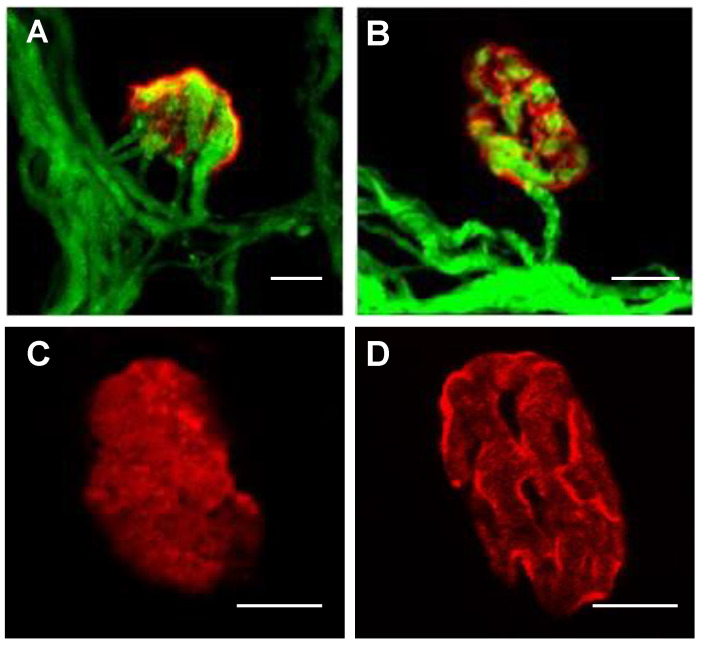
Confocal images of the postnatal innervation and morphological maturation of the postsynaptic nAChR clusters in P9 Thy1-YFP-16 normal mice. NMJ show the axons in green (mice express spectral variants of GFP, yellow-YFP) and the postsynaptic nicotinic acetylcholine receptor (nAChR) clusters stained in red with TRITC-α- BTX. (**A**), polyinnervated NMJ; (**B**), mature NMJ innervated by one axon; (**C**), a representative immature postsynaptic uniform cluster with no differentiated “holes”—endplate areas without receptors- and individualized gutters and in (**D**), a mature nAChR cluster. Scale bar: 10 μm.

The animals received an injection (50 μL) from P5 to P8 and the LAL muscles were dissected on day P9. Then, after fixation, muscles were incubated with tetramethylrhodamine conjugated α-bungarotoxin (TRITC-α-BTX, 1 h at room temperature; 1:800 dilution of 1 µg/mL; Molecular Probes, Eugene, OR, USA). Analysis of innervation and mAChR maturation were made by using confocal microscopy (Figure 1). To see the effect of the treatments on the nerve terminals, the number of axons innervating each nAChR receptor cluster were counted. The NMJs were classified in monoinnervated or polyinnervated (innervated by two or more terminal axons). At the same time, the percentage of immature nAChR clusters was defined as the uniform, density-homogeneous nAChR oval plaques, without inhomogeneities in the receptor density or the presence of initial gutters [12,13,14]. Percentages of multiple innervation and immature nAChR receptor clusters were assessed by Fisher’s test and Bonferroni correction. Twelve muscles from six mice were studied for each condition. A minimum of 100 NMJs per muscle were analyzed. The criterion for statistical significance was *p* < 0.05. The data are presented as percentages of NMJ ± SD. * *p* < 0.05, ** *p* < 0.01, *** *p* < 0.005.Table 2 and Table 3 show the comparison of the effects of specific PKA and PKC activators and inhibitors on the percentage of immature multiinnervated synapses and immature postsynaptic nAChR clusters (nAChR with uniform oval plaque).

## 2. Results and Conclusions

Firstly, we compared PKA activators with PKC inhibitors (both situations delayed synaptic maturation) and we found no difference in either number of axons per synapse or in the morphological postsynaptic maturation (in this last case, only when general PKC inhibitors are used). However, the specific block of cPKCβI or nPKCε with inhibitory peptides (βIV_5–3_ and εV_1–2_ respectively) results in no postsynaptic alteration and this is a significatively different situation from PKA stimulation showing the specific presynaptic site of action of these PKC isoforms. In parallel, when comparing PKA inhibitors with PKC activators (the trend of both situations is to accelerate maturation), no difference regarding axonal elimination was found. However, some differences in the nAChR clusters maturation emerged because isoform non-selective PKC activators (mainly PMA) strongly accelerates postsynaptic maturation, suggesting the involvement of another PKC isoform at this site.

**Table 2:** Percentage of multiinervation in P9 NMJs.

**Multiinervation** **(%)**				**PKC**								
				Control	Activator				Inhibitor			
				**PBS** **P9**	**BRY- 1** **(1 nM)**	**PMA** **(10 nM)**	**dPPA** **(0.2 µg/mL)**	**FR 236924** **(100 nM)**	**CaC** **(200 nM)**	**Che** **(1 μM)**	**β** **IV_5–3_** **(10 μM)**	**εV_1–2_** **(10 μM)**
				41.78 ± 5.61	29.16 ± 5.43	32.7 ± 2.68	26.69 ± 3.02	30.59 ± 6.32	77.00 ± 6.11	68.17 ± 8.21	73.64 ± 4.54	79.98 ± 9.44
**PKA**	**Control**	**PBS** **P9**	41.78 ± 5.61	-	*	*	*	*	***	***	***	***
	Activator	**Sp8Br** **(10 μM)**	77.31 ± 5.13	***	***	***	***	***	ns	ns	ns	ns
	Inhibitor	**H89** **(5 μM)**	32.11 ± 2.53	***	ns	ns	ns	ns	***	***	***	***
		**Rp8-Br** **(100 μM)**	35.03 ± 4.76	ns	ns	ns	ns	ns	***	***	***	***
		**Rp8-Br** **(300 μM)**	31.05 ± 3.37	*	ns	ns	ns	ns	***	***	***	***
		**Rp-cAMPs** **(100 μM)**	32.04 ± 6.28	ns	ns	ns	ns	ns	***	***	***	***
		**Rp-cAMPs** **(300 μM)**	39.24 ± 5.30	ns	ns	ns	ns	ns	***	***	***	***

PKA activators and inhibitors were cross compared with PKC activators and inhibitors. Fisher’s test and Bonferroni correction. The data are presented as percentages of NMJ ± SD. * *p* < 0.05, *** *p* < 0.005. *PKC Activators*: Bryostatine-1 (BRY); phorbol 12-myristate 13-acetate (PMA); 12-deoxyphorbol-13-phenylacetate-20-acetate (dPPA, βI PKC selective activator); 2-((2-Pentylcyclopropyl-methyl) cyclopropaneoctanoic acid (FR236924, ε PKC selective activator); *PKC Inhibitors*: Calphostin C (CaC); Chelerytrine (Che); Peptide βIV _5–3_ (βI PKC selective inhibitor); Peptide εV_1–2_ (ε PKC selective inhibitor); *PKA Inhibitors*: N-[2-((p-Bromocinnamyl)amino)ethyl]-5-isoquinolinesulphonamide, 2HCl (H89); 8-Bromoadenosine-3′,5′-cyclic monophosphorothioate, Rp-isomer sodium salt (Rp8-Br, RI-PKA selective,); Adenosine-3′,5′-cyclic monophosphorothioate, Rp-isomer sodium salt (Rp cAMPs, RII-PKA selective). *PKA Activators*: Adenosine 3′,5′ cyclic Monophosphorothioate,8-Bromo-, Sp-Isomer, Sodium Salt (Sp8Br).

**Table 3:** Percentage of immature AChR postsynaptic clusters in P9 NMJs.

**Immature clusters** **(%)**				**PKC**								
				Control	Activator				Inhibitor			
				**PBS** **P9**	**BRY-1** **(1 nM)**	**PMA** **(10 nM)**	**dPPA** **(0.2 µg/mL)**	**FR 236924** **(100 nM)**	**CaC** **(200 nM)**	**Che** **(1 μM)**	**β** **IV_5–3_** **(10 μM)**	**εV_1–2_** **(10 μM)**
				9.31 ± 1.73	4.76 ± 1.16	1.65 ± 0.47	8.79 ± 1.37	5.92 ± 1.25	23.30 ± 4.85	16.96 ± 5.61	11.10 ± 5.76	7.14 ± 3.36
**PKA**	**Control**	**PBS** **P9**	9.31 ± 1.73	-	***	***	ns	ns	***	**	ns	ns
	Activator	**Sp8Br** **(10 μM)**	28.57± 6.04	***	***	***	***	***	ns	ns	**	***
	Inhibitor	**H89** **(5 μM)**	5.60 ± 2.12	ns	ns	***	ns	ns	***	***	*	ns
		**Rp8-Br** **(100 μM)**	10.05 ± 2.59	ns	ns	***	ns	ns	***	ns	ns	ns
		**Rp8-Br** **(300 μM)**	16.74 ± 1.09	*	***	**	*	**	ns	ns	ns	**
		**Rp-cAMPs** **(100 μM)**	4.70 ± 1.97	*	ns	***	ns	ns	***	**	**	ns
		**Rp cAMPs** **(300 μM)**	8.57 ± 3.26	ns	ns	***	ns	ns	***	*	ns	ns

PKA activators and inhibitors were cross compared with PKC activators and inhibitors. Fisher’s test and Bonferroni correction. The data are presented as percentages of NMJ ± SD. * *p* < 0.05, ** *p* < 0.01, *** *p* < 0.005. See the Table 2 for meaning of drugs abbreviation.

Second, when comparing the effects of PKA and PKC activators, a significant difference is always observed when looking both for the number of axons per synapse and for the postsynaptic maturation (the significance is always *p* < 0.005, Fisher’s test) indicating the complementarity of the kinases. Finally, when comparing the effects of PKA and PKC inhibitors a significant difference is always observed when looking for the number of axons per synapse (the significance is also always *p* < 0.005). However, the comparisons in relation with the postsynaptic maturation show some complexity. Specifically, as stated above, PKA inhibition (with H-89 or some specific RI or RII blockers) barely induces a small change on the postsynaptic clusters, indicating that pharmacologic PKA inhibition above the physiological situation cannot be increased. On the other hand, and similarly, the specific blockers of cPKCβI or nPKCε do not affect nAChRs clusters because of their presynaptic site of action. Thus, it seems that, in the synapse elimination process at the considered developmental period (P9), PKCs may have a significative role in determining maturation as their experimental inhibition strongly affects the process. However, endogenous PKA activity seems to be inhibited during the synapse elimination process because the additional pharmacological inhibition only results in a presynaptic change. Thus, PKA would be not much further additionally inhibited and PKC would be not much further activated, suggesting there is a well balanced effect between them (a similar level of PKA inhibition and PKC stimulation resulting in synapse maturation). Altogether, these results suggest a well stablished balance between PKC and PKA activity that allows the optimal NMJ maturation progression, being PKC more active than PKA during it.

In summary, there are no significant differences exist between the effects of PKA activators and PKC inhibitors or PKA inhibitors and PKC activators on the rate of developmental axon loss rate, indicating the complementarity of the kinases. Indeed, a similar level of PKA inhibition and PKC potentiation (mainly of the cPKCβI and nPKCε isoforms) would be required during development. On the other hand, the kinase effect in the postsynaptic clusters maturation follows the same pattern of PKA/PKC interaction as in the axons despite that without the involvement of cPKCβI and nPKCε isoforms. These detailed statistical results strongly reinforce the previously published data, indicate a direct reciprocal involvement of the PKA and PKC isoforms, and suggest a complementary or cooperative work of them.

To go further into the issue, the following question can be asked: What can be the precise differential involvement of PKA inhibition and PKC activation in nerve terminals in different stages of competition and elimination or strengthening? We should analyze the hypothesis that this particular configuration of kinase’s activity in supernumerary axons is done to be eliminated.

## Figures and Tables

**Table 1:** PKC and PKA activators and inhibitors and their targets.

	**ACTIVATOR**	**TARGET**
PKC	BRY	PKC activator
	PMA	PKC activator
	dPPA	βI PKC selective activator
	FR236924	ε PKC selective activator
PKA	Sp8Br	PKA activator
	**INHIBITOR**	**TARGET**
PKC	CaC	PKC pan inhibitor
	Che	PKC pan inhibitor
	Peptide βIV _5-3_	β-PKC selective inhibitor
	Peptide εV _1-2_	ε-PKC selective inhibitor
PKA	H-89	PKA inhibitor
	Rp8-Br	RI-PKA selective inhibitor
	Rp-cAMPs	RII-PKA selective inhibitor

## Data Availability

We think that our data are not appropriate for the available repository database in neuroscience.

## References

[1] O’Brien R.A., Ostberg A.J., Vrbová G. (1978). Observations on the elimination of polyneuronal innervation in developing mammalian skeletal muscle. J. Physiol..

[2] Redfern P.A. (1970). Neuromuscular transmission in new-born rats. J. Physiol..

[3] Smith I.W., Mikesh M., Lee Y., Thompson W.J. (2013). Terminal Schwann cells participate in the competition underlying neuromuscular synapse elimination. J. Neurosci..

[4] Lee Y. (2020). Developmental neuromuscular synapse elimination: Activity-dependence and potential downstream effector mechanisms. Neurosci. Lett..

[5] Favero M., Cangiano A., Busetto G. (2014). The timing of activity is a regulatory signal during development of neural connections. J. Mol. Neurosci..

[6] Tomàs J., Garcia N., Lanuza M.A., Santafé M.M., Tomàs M., Nadal L., Hurtado E., Simó A., Cilleros V. (2017). Presynaptic membrane receptors modulate ACh release, axonal competition and synapse elimination during neuromuscular junction development. Front. Mol. Neurosci..

[7] Tomàs J., Garcia N., Lanuza M.A., Santafé M.M., Tomàs M., Nadal L., Hurtado E., Simó-Ollé A., Cilleros-Mañé V., Just-Borràs L. (2018). Adenosine receptors in developing and adult mouse neuromuscular junctions and functional links with other metabotropic receptor pathways. Front. Pharm..

[8] Nadal L., Garcia N., Hurtado E., Simó A., Tomàs M., Lanuza M.A., Cilleros V., Tomàs J. (2017). Presynaptic muscarinic acetylcholine receptors and TrkB receptor cooperate in the elimination of redundant motor nerve terminals during development. Front. Aging Neurosci..

[9] Garcia N., Balañà C., Lanuza M.A., Tomàs M., Cilleros-Mañé V., Just-Borràs L., Tomàs J. (2019). Opposed Actions of PKA Isozymes (RI and RII) and PKC Isoforms (cPKCβI and nPKCε) in Neuromuscular Developmental Synapse Elimination. Cells.

[10] Lanuza M.A., Garcia N., Santafe M., Nelson P.G., Fenoll-Brunet M.R., Tomas J. (2001). Pertussis toxin-sensitive G-protein and protein kinase C activity are involved in normal synapse elimination in the neonatal rat muscle. J. Neurosci. Res..

[11] Nadal L., Garcia N., Hurtado E., Simó A., Tomàs M., Lanuza M.A., Santafé M., Tomàs J. (2016). Presynaptic muscarinic acetylcholine autoreceptors (M1, M2 and M4 subtypes), adenosine receptors (A1 and A2A) and tropomyosin-related kinase B receptor (TrkB) modulate the developmental synapse elimination process at the neuromuscular junction. Mol. Brain.

[12] Steinbach J.H. (1981). Developmental changes in acetylcholine receptor aggregates at rat skeletal neuromuscular junctions. Dev. Biol..

[13] Slater C.R. (1982). Postnatal maturation of nerve-muscle junctions in hindlimb muscles of the mouse. Dev. Biol..

[14] Lanuza M.A., Garcia N., Santafé M., González C.M., Alonso I., Nelson P.G., Tomàs J. (2002). Pre- and postsynaptic maturation of the neuromuscular junction during neonatal synapse elimination depends on protein kinase C. J. Neurosci. Res..

